# Knowledge and attitude for overactive bladder care among women: development and measurement

**DOI:** 10.1186/s12894-018-0371-2

**Published:** 2018-06-05

**Authors:** Sumedha Chhatre, Diane K. Newman, Alan J. Wein, Ashlie E. Jefferson, J. Sanford Schwartz, Ravishankar Jayadevappa

**Affiliations:** 1 Department of Psychiatry, Perelman School of Medicine, University of Pennsylvania, 3535 Market St., Suite 4051, Philadelphia, PA 19104 USA; 2 Division of Urology, Department of Surgery, Perelman School of Medicine, University of Pennsylvania, Philadelphia, PA 19104 USA; 3 Department of Medicine, Perelman School of Medicine, University of Pennsylvania, Philadelphia, PA 19104 USA; 4 Departments of Medicine and Health Management, Leonard Davis Institute of Health Economics, University of Pennsylvania, Perelman School of Medicine and Wharton School of Business, Philadelphia, PA 19104 USA; 5 Departments of Medicine and Surgery, Divisions of Geriatrics and Urology, Perelman School of Medicine Leonard Davis Institute of Health Economics, University of Pennsylvania, Philadelphia, PA 19104 USA

**Keywords:** Overactive bladder, Women, Knowledge and attitude, Treatment uptake

## Abstract

**Background:**

Overactive bladder (OAB) affects millions of women. It is important to assess knowledge and attitude in affected patients. The study objective was to develop surveys to assess OAB knowledge and OAB related attitude, and its association with OAB treatment status.

**Methods:**

Systematic literature review and qualitative analysis of patient and provider focus groups helped identify OAB knowledge and attitude survey items. We determined psychometric properties of the two surveys in a cross-sectional sample of 104 women, 27% of whom had received OAB treatment.

**Results:**

The OAB-knowledge survey consisted of 16 items and 3 condition-related concepts: perception of OAB; cause and information; and signs of OAB. The OAB-attitude survey consisted of 16 items and its concepts were treatment seeking; decision-making and effects. Both surveys demonstrated good construct validity and test-retest reliability ((≥ 0.60). In the cross-sectional validation sample, OAB-knowledge and attitude discriminated between those with different levels of ICIQ-UI scores. We observed some difference in the OAB knowledge, OAB attitude, and severity of symptoms between those treated for OAB vs. treatment naive.

**Conclusions:**

OAB knowledge and attitude surveys provide a novel tool to assess OAB domains in women. Though we did not find statistical significance in OAB knowledge and attitude scores across treatment status, they may be potentially modifiable factors that affect OAB treatment uptake and treatment compliance. Refinement of these surveys in diverse sub-populations is necessary. Our study provides effect sizes for OAB knowledge and attitude. These effect sizes can help development of fully powered trials to study the association between OAB knowledge and attitude, type and length of treatment, treatment compliance, and quality of life, leading to interventions for enhancing OAB care.

**Electronic supplementary material:**

The online version of this article (10.1186/s12894-018-0371-2) contains supplementary material, which is available to authorized users.

## Background

A common health concern for men and women of all ages, overactive bladder (OAB) is defined by the International Continence Society as urgency, with or without urge urinary incontinence (UI), frequently accompanied with frequency and nocturia in the absence of proven infection or other obvious pathology [1]. An embarrassing and debilitating condition with substantial health and economic consequences [1–3], OAB affects nearly 17% of women in the US. Prevalence of OAB increases with age and among women aged 65 and older, the prevalence of OAB is approximately 30% [2–5].

The symptoms of OAB can have negative effects on quality of life and necessitate lifestyle changes [6–9]. The National Overactive Bladder Evaluation (NOBLE) Program data showed that OAB with or without urgency urinary incontinence was associated with significantly lower quality of life and quality of sleep, and higher depression, compared to controls [2]. Total cost related to OAB in the US was more than $12 billion in 2000 using the NOBLE survey data. These costs are comparable to those of osteoporosis and gynecological cancers [5]. However, many women with OAB suffer in silence without seeking care [1–9], while attempting to manage their symptoms by developing coping mechanisms.

### Conceptual framework

**Fig. 1:**
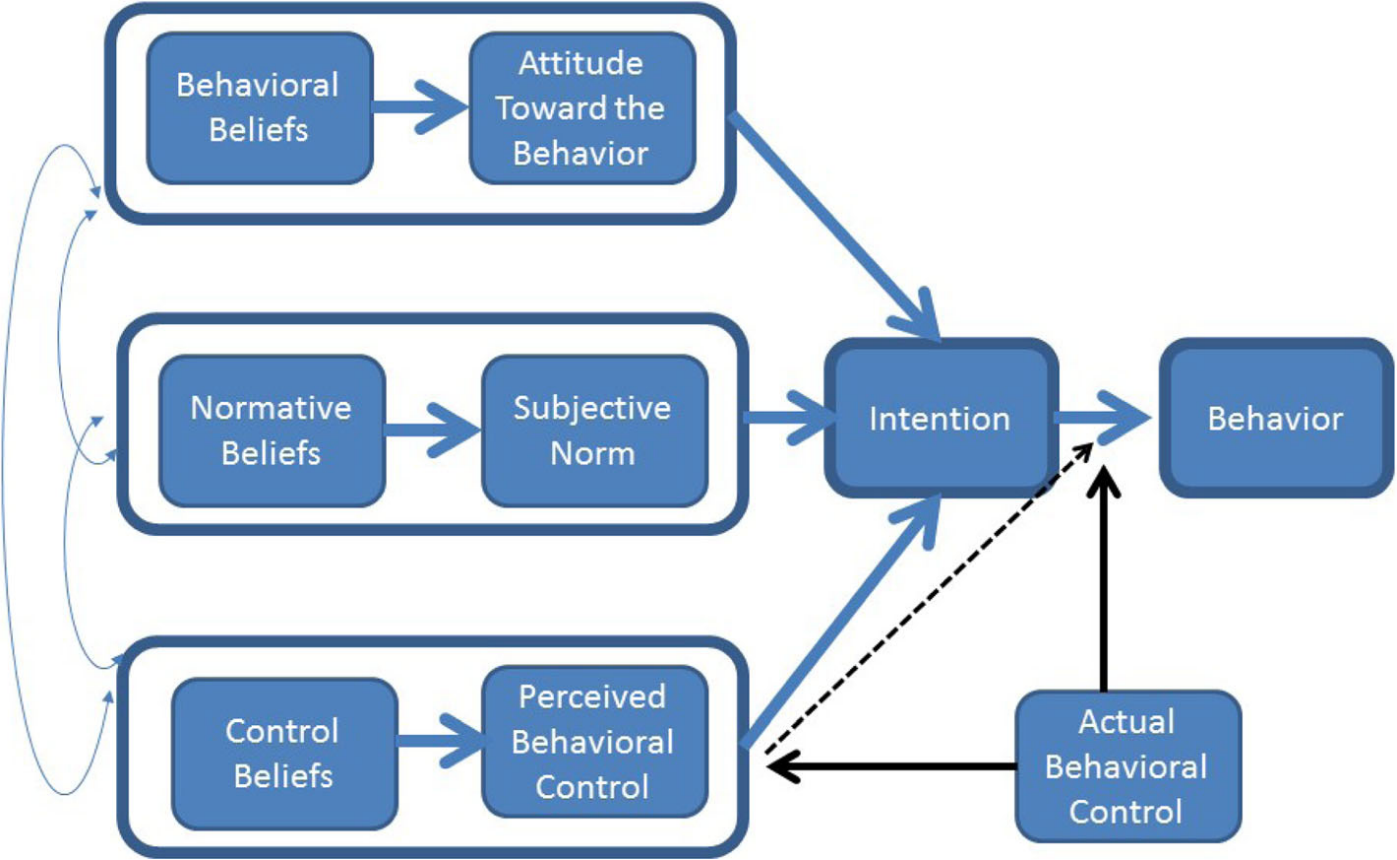
Conceptual Model-Theory of Planned Behavior (Ajzen, 1991)

Our study conceptual model is based on the Theory of Planned Behavior (TPB) [10]. The TPB posits that human behavior results from intentions, which in turn are driven by attitude toward behavior; subjective norms; and perceived behavioral control. Knowledge plays an important role in each of these. In this study, the focus is on the assessment of knowledge of and attitude towards OAB. This information, when linked with the observed behavior, can inform interventions to influence OAB care seeking behavior (Fig. 1).

Knowledge regarding OAB and attitudes towards seeking care for OAB can affect the uptake of OAB care [11]. Therefore, the objective of this study was to develop psychometrically sound surveys to assess knowledge and attitudes among adult women from a large academic, urban healthcare system. We also studied if OAB knowledge and attitude was associated with OAB treatment and if those with higher level of OAB knowledge had more positive attitude towards OAB care.

## Methods

In this study, we evaluated the knowledge and attitude among women with OAB. We used theory of TPB to postulate a conceptual framework of knowledge of and attitude toward OAB care. Two phases of our study were: (1) development of OAB knowledge and attitude items and surveys; and (2) psychometric testing of the surveys. The local Institutional Review Board approved the study.

### Survey development

In Phase 1, we conducted in-depth literature review, and patient and provider focus groups to determine OAB-knowledge and OAB-attitude survey items [12]. As OAB symptoms in women often are associated with perceived social stigma, we included the term “stigma” in our literature review. We also searched for OAB related surveys to expand our framework for knowledge and attitude measurement. We conducted a comprehensive review of the literature published in English from 1990 to 2014 using Medline, PUBMED, CINAHL, and EMBASE. A key term search strategy was employed using “Overactive bladder (urgency, frequency and urge urinary incontinence)”, “incontinence”, “nocturia”, “health related quality of life (HRQoL)”, “symptoms”, “attitude”, and “knowledge”.

Next, we conducted patient focus groups to explore their knowledge of OAB, experience of OAB symptoms and attitudes towards seeking OAB care. Prior to focus groups, we developed an interview guide with instructions, think-aloud exercises, and scripted probes. All focus group participants provided written consent. An experienced moderator led each focus group with assistance from co-facilitators (DK and RJ). Similarly, two provider focus groups yielded expert’s perceptions of OAB care. Focus group discussions were audio taped and transcribed. Two reviewers (SC and RJ) analyzed the transcribed text using NVivo software, a qualitative analysis program that allows coding and classification of text into major and minor concepts.

Informed by the literature review and focus groups, we assembled an initial pool of items for OAB-knowledge and OAB-attitude surveys. These items were reviewed by external and internal collaborators who gave feedback on the face validity (clarity and relevance) of the candidate items. The qualitative item review was initiated by developing draft OAB-knowledge and OAB-attitude surveys. We employed best practices [13–15] for creating new questions for the surveys, reviewing items to ensure that they: (1) did not exceed a sixth-grade reading level, as measured by the Flesch–Kincaid Grade Level standard; (2) minimized ambiguity or cognitive difficulty; (3) avoided multi-barreled questions; (4) were concisely and simply worded; and (5) were easy to translate into other languages.

### Statistical analysis

The internal reliability of the OAB knowledge and attitude surveys was assessed by Cronbach’s alpha (≥ 0.60) with items with low item-total correlations excluded. A random-effects, repeated measures analysis of variance was used to compute intra-class correlation coefficients (ICC) for the seven-day test–retest administration of the surveys. The minimum threshold for re-test was an ICC of 0.40 [12, 16]. We determined the number of participants required for test–retest reliability assessment based on a target of 0.80 (excellent) ICC for the pair of total scores on each measure against an ICC of 0.50 (fair). On this basis, we estimated that at least 21 participants would be needed [16]. Construct validity was evaluated by using confirmatory factor analysis; concurrent validity was evaluated by comparing the OAB knowledge and OAB attitude with ICIQ-UI scores. We also explored if OAB knowledge, OAB attitude and severity of symptoms (ICIQ-UI scores) varied by OAB treatment status.

## Results

### Literature review

From literature review we identified three preliminary OAB knowledge and attitude concepts: (1) OAB symptoms and treatment; (2) psychological symptoms (e.g., anxiety, depression, fear, frustration), and (3) social symptoms (e.g., disease stigma, intimacy and sexuality, embarrassment, diminished work productivity). We used these concepts and expert feedback to develop an initial conceptual model and focus group script.

### Focus group

Five focus group meetings of patients (total *n* = 62) were conducted. Two focus groups consisted of participants recruited from a database of women who failed prescreening for enrollment in a urinary incontinence prevention study. The third and fourth focus group consisted of women who received care from a large urban academic healthcare system. The fifth focus group consisted of female members of the Living Independently for Elders program. Not all focus group participants reported having OAB. Average length of each focus group was 45 min. In addition, two provider focus groups were conducted. One consisted of primary care physicians, geriatricians, and geriatric nurse practitioners. The other one was among providers of the Living Independently for Elders program.

Data collection continued until saturation occurred [13–15]. Content analysis was performed through open coding by the same investigator [13–15]. Peer review and consistency of the analysis occurred whereby another investigator independently analyzed transcripts using open coding and then crosschecked for discrepancies.

#### Item Development

Informed by literature review and focus groups, we created an initial pool of 18 and 19 items for OAB-knowledge and OAB-attitude surveys, respectively. OAB-knowledge items were related to three general themes: perception of OAB; cause of and information about OAB; and signs of OAB. OAB-attitude items were related to three general themes of treatment seeking, decision making and effects of OAB.

Field versions of the measures were constructed as follows. The OAB-knowledge items used a ‘yes’/‘no’ or ‘don’t know’; or ‘true’/‘false’ or ‘don’t know’ response format (each correct answer scored one and ‘don’t know’ and incorrect responses scored zero). Responses to the OAB-attitude surveys were anchored by a five-point Likert type scale (‘completely disagree’, ‘disagree’, ‘unsure’, ‘agree’, and ‘completely agree’). The order of the items was random. After eliminating the items that were duplicative, or unrelated to the constructs, we retained 16 items in each survey. Please see Additional file 1 for OAB knowledge and attitude surveys.

### Cross sectional sample characteristics

To validate the OAB-knowledge and OAB-attitude surveys, we contacted 244 women in total. Of these 170 were from a database of women who failed prescreening for enrollment in a urinary incontinence prevention study; and the rest were identified from the administrative database of a large urban academic healthcare system. Of these 244 women, 115 provided written consent and 104 completed the assessments. A research coordinator administered the International Consultation of Incontinence Modular Questionnaire – Urinary Incontinence (ICIQ-UI) form, OAB-knowledge, and OAB-attitude surveys over telephone. Seven-day test–retest reliability was assessed among 21 randomly selected participants. More than 90% of the participants were between the age of 56 and 75, about three-quarters were white, a large proportion were college educated, about half were retired and 60% were married.

In Tables 1 and 2, show the response distribution for the OAB-knowledge and OAB-attitude surveys. The average OAB knowledge score was 9.1 (±1.9). The possible range is 0 to 16 and higher score represents better OAB knowledge. The average attitude score was 42.8 (±6.6). The possible range for attitude score is 16–80, and lower score represents more positive attitude towards OAB care. Almost all participants had heard of OAB and were aware of treatment options for OAB (98%). Most agreed that treatment benefits outweigh its costs (76%), Slightly more than half said they were very likely to seek treatment for OAB (54%). The women in our sample were very likely to seek behavioral treatment (such as such as Kegel exercise) for OAB than pharmacological treatment (72% vs. 8%). A large proportion of women thought that OAB will very likely cause stigma (47%) and affect quality of life (40%).

**Table 1:** Overactive Bladder (OAB) Knowledge (*n* = 104)

Have you heard of overactive bladder syndrome (OAB), which includes urinary urgency, frequency, and nocturia with or without urgency incontinence? (% yes)	98.1		
Knowledge items (%)	True	False	Don’t know
OAB is a natural aging process	52.9	43.3	3.9
OAB happens mostly in women	59.6	34.6	5.8
There are no treatments for OAB	6.7	92.3	0.96
OAB is related to childbirth	44.2	53.9	1.9
My doctor can tell me if I have OAB	68.3	30.8	0.96
Any sickness can cause OAB	39.4	54.8	5.8
OAB has specific symptoms	91.4	7.7	0.96
Treatments for OAB have many side effects	27.9	70.2	1.9
OAB is a chronic disease	42.3	55.8	1.9
OAB can go away on its own	17.3	80.8	1.9
I can get all information about OAB from internet	34.6	63.5	1.9
Pharmacological (i., e drug) treatment is available for OAB	86.5	11.5	1.9
OAB is caused by an enlarged prostate	42.3	52.9	4.8
Insurance does not cover treatment for OAB	12.5	77.9	9.6
OAB medication is too expensive	23.1	63.5	13.5
The benefit of OAB medication is worth the cost	76.0	17.3	6.7
Total OAB knowledge score (mean, std)	9.1 (±1.9)

**Table 2:** Overactive Bladder (OAB) Attitude (*n* = 104)

Attitude items (%)	Very likely	Somewhat likely	Neutral	Somewhat unlikely	Very Unlikely
How likely are you to ask your doctor about OAB?	45.2	24.0	4.8	14.4	11.5
If you have OAB, how likely are you to seek treatment?	53.9	28.9	3.9	6.3	6.3
How likely will you research about OAB?	52.9	29.8	5.8	3.9	7.7
How likely are you to seek pharmacological (drug) treatment for OAB?	7.7	33.7	10.6	25.9	22.2
How likely are you to seek behavioral (such as Kegel exercise) treatment for OAB?	72.1	23.1	1.9	0.96	1.9
How likely are you to seek surgery for OAB?	3.9	9.6	11.5	25.0	50.0
How likely will you seek other medical treatment for OAB? (e.g.acupuncture, yoga, meditation, herbal medicine, etc.)	32.7	34.6	14.4	13.5	4.8
How likely will side effects of a treatment affect your decision to seek treatment?	58.7	29.8	6.7	3.9	0.96
What is the likelihood OAB is causing you embarrassment?	47.1	31.7	4.8	7.7	8.9
How likely will OAB affect your quality of life?	40.4	30.8	7.7	13.5	7.7
How likely will cost affect your decision to seek treatment of OAB	10.6	27.9	9.6	29.0	27.9
What is the likelihood that wearing pads for protection will bother you?	17.3	25.9	12.5	20.2	24.0
How likely will you simply ignore the OAB problem?	5.8	11.5	2.9	26.9	52.9
How likely will you be to support a public health campaign about OAB awareness?	65.4	25.9	4.8	2.9	0.96
How likely would you be to discuss OAB with friends and family?	49.0	37.5	3.9	6.7	2.9
How likely would you be to continue with OAB treatment despite side effects?	11.5	27.9	22.1	25.0	13.5
Total OAB attitude score (mean, std)	42.8 (±6.6)				

The mean ICIQ score was 6.6 (SD 3.3). The total ICIQ score ranges from 0 to 21 and a higher score indicates greater severity of symptoms (Table 3). Factor analysis yielded a three-factor solution for the obliquely rotated factor pattern for the surveys. We identified three OAB knowledge factors - perception of OAB, cause and information, and signs of OAB; and three OAB-attitude factors - treatment seeking, decision-making, and effects of OAB. Inter-correlations of the sub-scales supported the validity of a single, higher order OAB-knowledge and OAB-attitude scales, which was not refuted by the confirmatory factor analysis, thus helping establish the construct validity of the surveys.

**Table 3:** ICIQ UI Short Form (*n* = 104)

ICIQ items	
How often do you leak urine? (%)	
Never	26.9
About once a week or less	41.4
2–3 times a day	20.2
About once a day	7.7
Several times a day	3.9
All the time	0.0
How much urine do you think usually leaks (whether you wear protection or not)? (%)	
None	22.1
A small amount	69.2
A moderate amount	8.7
A large amount	0.0
Overall, on a scale from zero to 10, where zero is “not at all” and 10 is a “great deal”, How much does leaking urine interfere with your everyday life?	Mean = 1.8 (±2.3)
Total ICIQ score	Mean = 6.6 (±3.3)
Does urine leak (% yes)	
Before you go to the toilet?	60.6
When you cough or sneeze?	51.9
When you are asleep?	7.7
When you are physically active or exercising?	27.9
When you have finished urinating and are dressed?	14.4
For no obvious reason	16.4
All the time?	0.96
Never – urine does not leak	22.2

The total scores of the OAB-knowledge and OAB-attitude surveys demonstrated statistically significant correlations with the ICIQ total scores, 0.3231 (*p* = 0.0009) and − 0.4454 (*p* < 0.0001), respectively. The OAB-knowledge and attitude scores varied significantly between patients with low ICIQ scores (0–7), moderate ICIQ scores (8–14) and high ICIQ scores (15–21). The mean knowledge scores were 8.6 (±1.6), 9.4 (±1.0) and 10.8 (±0.5) for the low, moderate and high ICIQ score groups, respectively. Similarly, the mean attitude scores were 44.8 (±5.2), 42.0 (±6.9) and 33.2 (±3.3) for the low, moderate and high ICIQ score groups, respectively. These associations between OAB knowledge scores, OAB attitude scores and ICIQ scores helped establish the concurrent validity of the surveys. Finally, all OAB-knowledge and OAB-attitude sub-scales demonstrated high test-retest reliability, with Cronbach’s α for each exceeding our a priori threshold of ≥0.70, indicating good test-retest reliability of the surveys.

Seventy-six (73.1%) women were treatment naïve whereas 28 (26.9%) had either pharmacological or behavioral treatment for OAB. We did not obtain data on treatment length or treatment compliance. The mean OAB knowledge score was 9.0 (SD 8.7) for the treatment naïve group, and 9.3 (SD 8.4) for those with treatment (*p* = 0.6446; effect size 0.10). Mean attitude score was 42.6 (SD 6.6) for the treatment naïve group, and 43.3 (SD 6.4) for those with treatment (*p* = 0.5956; effect size 0.12). Finally, the severity of symptoms as measured by ICIQ-UI was 5.9 (Sd 3.3) for the treatment group, compared to 7.0 (SD 3.6) for the treatment naïve group (*p* = 0.1812; effect size 0.29).

## Discussion

Our OAB-knowledge and attitude surveys are an important step in assessment of the association between OAB knowledge, attitude, and treatment uptake and treatment compliance. We observed some difference in the OAB knowledge, OAB attitude, and severity of symptoms across treatment status. Though we did not find statistical significance in these differences, they may be potentially modifiable factors that affect OAB treatment uptake and treatment compliance.

Approximately 33 million Americans (16.5% of the U.S. population) experience OAB [1–9]. The genesis of symptoms of OAB commonly is multi-factorial, and thus multimodal therapy that includes pharmacologic and non-pharmacologic interventions may be necessary. However, OAB remains underdiagnosed and undertreated. A Finnish study analyzed the effects of frequency of urinary urgency and urge urinary incontinence on symptom-related bother, HRQoL, and the clinically meaningful prevalence of overactive bladder [17]. The study consisted of 6000 subjects (age 18–79 years) randomly identified from the Finnish population register in 2003–2004, with 62.4% responding. Urgency was reported by more than half, whereas urinary incontinence was reported by 25.7% of women. It was concluded that increased severity of urgency and urinary incontinence is associated with a statistically significant and clinically important decrease in HRQoL.

Older age, genetics, female sex, pregnancy, childbirth, stress and extreme physical activity are generally perceived as causes of OAB. Symptoms are under-reported in women, mainly due to limited understanding of or appreciation of the morbidity of the condition. Women tend to think of OAB as normal part of aging and develop coping mechanisms, rather than seeking care [3]. The cultural model constructed by women differs significantly from the professional model that emerged from media representations of female urinary incontinence [18]. Despite a wide variety of treatments for OAB, many women choose not to seek care [19–21]. Further exploration of the “disconnect” between the experiences of women who live with urinary incontinence and common public views of female urinary incontinence may lead to an increased appreciation and understanding of these issues [22]. A mixed-methods study conducted needs assessment of OAB patients. Significant time-gap was noted between the onset of OAB symptoms and diagnosis of OAB. This indicates need for better OAB screening and diagnosis [23].

Our results make important contribution to the existing research on OAB. Our cohort of women thought that OAB happens mostly in women. A large proportion had heard of OAB and was aware of treatments options for OAB. Also, most agreed that treatment benefits outweigh its costs, however, only half said they were very likely to seek treatment for OAB. The women in our sample were more likely to seek behavioral treatment or self-management for OAB than pharmacological treatment. A large proportion of women thought that OAB will likely cause stigma and affect quality of life.

We note following limitations to the study. First limitation is selection bias as participants were either self-selected to enroll in a urinary incontinence prevention study or were selected from a large urban academic healthcare system database. Hence, symptom severity of women in our cohort may not be representative of the general population of women with OAB. Participants were recruited via telephone during business hours and some of the focus groups also were held during those hours. As a result, the majority of those participated were retired, and older. Therefore, we may not have fully captured the experiences of younger adult women with OAB or of men with OAB. Additionally, the ICIQ-UI survey is not validated for telephone administration. Another limitation is that of discrepancies and biases in qualitative data analysis. To minimize this, two investigators analyzed the data separately. Finally, we did not collect information about length of OAB treatment, or treatment compliance that may affect overall association between treatment status, OAB knowledge and OAB attitude. While usually not life threatening, OAB has a significant negative impact on quality of life and can adversely affect self-esteem, resulting in embarrassment, diminished social relations, sexual satisfaction, professional and social life interactions, and overall wellbeing [22–28].

Our surveys must be refined for assessment among different subpopulations based on age, gender, and functional status. Knowledge plays an important role in shaping a person’s attitude toward behavior, subjective norms, and perceived behavioral control. Our study provides effect sizes for OAB knowledge, OAB attitude and ICI UI scores. These effect sizes can form the basis for developing a fully powered trial to study the association between OAB knowledge and attitude, type and length of treatment, treatment compliance, and quality of life, leading to interventions for enhancing OAB care.

## Additional file

Additional file 1: Appendix- OAB knowledge and attitude surveys. (DOCX 25 kb)
