# Supplementary material for: Knowledge and attitude for overactive bladder care among women: development and measurement
Source: BMC Urol. 2018 Jun 5;18:56. doi: 10.1186/s12894-018-0371-2 (PMC5987448; doi:10.1186/s12894-018-0371-2)
Supplement: Supplementary file 1 — Appendix- OAB knowledge and attitude surveys. (DOCX 25 kb) [file 12894_2018_371_MOESM1_ESM.docx]

**PART I: DEMOGRAPHICS**

(1a) **What is your age group**?

□ Younger than 45 □ 46-55 □ 56-65

□ 66-75 □ 76-85 □86 or older □ Declined to answer

(1b) **What is your gender?**

□ Male □ Female □ Other _____________ □ Declined to answer

(1c) **What is your race/ethnicity?**

□ White

□ Black or African American

□ American Indian or Alaskan Native

□ Asian

□ Native Hawaiian or Pacific Islander

□ Spanish, Latino, or Hispanic

□ Mixed or other, please specify_________________________________

□ Declined to answer

(1d) **what is your level of education?**

□ Less than high school □ High school graduate □ Some college

□ College graduate □ Graduate degree

□ Declined to answer

(1e) Are you employed…….

□ Full time □Part time □Unemployed □ Retired □ Declined to answer

**PART II: KNOWLEDGE OF OVER ACTIVE BLADDER SYNDROME**

The next questions are about a bladder condition called: overactive bladder or OAB.

Persons who have OAB report going to the bathroom more often, having strong urges that cause you to rush to the bathroom, and you may or may not leak urine when you do not want to.

I would like to ask you some questions about OAB

(2a**) Have you heard of** **OAB, which can include urinary urgency, frequency, and waking up at night to urinate?**

□ Yes (continue) □ No (go to part III)

**Please answer True or False to the following questions:**

(2b) **OAB is a natural aging process**. □ True □ False

(2c) **OAB happens mostly in women**. □ True □ False

(2d) N**o treatments are available for OAB symptoms**. □ True □ False

(2e) **OAB is related to childbirth.** □ True □ False

(2f) **Your doctor can tell you if you have OAB.** □ True □ False

(2g) **Any sickness can cause** **OAB.** □ True □ False

(2h) **OAB has specific symptoms**. □ True □ False

(2i) **Treatments for OAB have many side effects.** □ True □ False

(2j) **OAB is a chronic disease**. □ True □ False

(2k) **OAB can go away on its own.** □ True □ False

(2l) **You can get all information about** **OAB from the internet.** □ True □ False

(2m) Drug **treatment is available for** **OAB.** □ True □ False

(2n) **OAB is caused by an large prostate**. □ True □ False

(2o) **Insurance does not cover treatment for OAB**. □ True □ False

(2n) **OAB medication is too expensive**. □ True □ False

(2o) **The benefit of OAB medication is worth the cost**. □ True □ False

**PART III: ATTITUDE TOWARDS OAB**

(3a) **How likely are you to ask your doctor about OAB?**

□ Very likely □Somewhat likely □ Neutral □Somewhat unlikely □ Very unlikely

(3b) **If you have OAB, how likely are you to seek treatment?**

□ Very likely □Somewhat likely □ Neutral □Somewhat unlikely □ Very unlikely

(3c) **How likely are you to research OAB on your own?**

□ Very likely □Somewhat likely □ Neutral □Somewhat unlikely □ Very unlikely

(3d) **How likely are you to seek drug treatment for OAB?**

□ Very likely □Somewhat likely □ Neutral □Somewhat unlikely □ Very unlikely

(3e) **How likely are you to seek behavioral treatment, such as Kegel exercises, for OAB?**

□ Very likely □Somewhat likely □ Neutral □Somewhat unlikely □ Very unlikely

(3f) **How likely are you to seek surgery for OAB?**

□ Very likely □Somewhat likely □ Neutral □Somewhat unlikely □ Very unlikely

(3g) **How likely would you be to seek other medical treatment for OAB?** (Examples are acupuncture, yoga, meditation, herbal medicine, etc.)

□ Very likely □Somewhat likely □ Neutral □Somewhat unlikely □ Very unlikely

(3h) **How likely will side effects of a treatment affect your decision to seek treatment?**

□ Very likely □Somewhat likely □ Neutral □Somewhat unlikely □ Very unlikely

(3i) **What is the likelihood of OAB causing you embarrassment?**

□ Very likely □Somewhat likely □ Neutral □Somewhat unlikely □ Very unlikely

(3j) **How likely will OAB affect your quality of life – your routine daily life?**

□ Very likely □Somewhat likely □ Neutral □Somewhat unlikely □ Very unlikely

(3k) **How likely will cost affect your decision to seek treatment of OAB?**

□ Very likely □Somewhat likely □ Neutral □Somewhat unlikely □ Very unlikely

(3l) **What is the likelihood that wearing pads for protection will bother you?**

□ Very likely □Somewhat likely □ Neutral □Somewhat unlikely □ Very unlikely

(3m) **How likely would you be to simply ignore the OAB problem?**

□ Very likely □Somewhat likely □ Neutral □Somewhat unlikely □ Very unlikely

(3n) **How likely would you be to support a public health campaign about OAB awareness?**

□ Very likely □Somewhat likely □ Neutral □Somewhat unlikely □ Very unlikely

(3o) **How likely would you be to discuss OAB with friends and family?**

□ Very likely □Somewhat likely □ Neutral □Somewhat unlikely □ Very unlikely

(3p) **How likely would you be to continue with OAB treatment despite side effects?**

□ Very likely □Somewhat likely □ Neutral □Somewhat unlikely □ Very unlikely
